# Report of the Pennsylvania Association of Dental Surgeons

**Published:** 1900-04

**Authors:** Theodore F. Chupein


ARTICLE II.

### REPORT OF THE PENNSYLVANIA ASSOCIATION OF DENTAL SURGEONS.

THEODORE F. CHUPEIN.

The painless removal of the dental pulp, by means of powdered cocain, applied directly to that organ on an alcohol-soaked piece of spunk, charged with anesthetic, and subsequently pressed upon with a ball burnisher, over a piece of unvulcanized rubber, so extensively of late spoken of in the dental journals, was referred to by Dr. Chupein.

Dr. Wessels had tried this plan on several occasions, but in a somewhat different manner. Instead of using only alcohol, he made a menstruum of five parts of alcohol and one part of formaldehide. Into this he dipped his spunk and applied to the exposed nerves, bringing increased pressure on the unvulcanized rubber as much as the patient could stand without discomfort.

When asked why he used the formaldehide, he stated that this material toughened the nerve as well as caused it to shrink, and left it in a better condition for the barks of the nerve-extractor to engage themselves into it, thereby making its removal easier.

He had not, in this, been always successful, as he believed if the nerve was in a hypertrophied condition, it would refuse to be anesthetized.

On the subject of the employment of arsenical paste and nerve fiber for devitalization, Dr. Chupein spoke of the greater safety of the latter over the former, yet could not speak of its effectiveness. With nerve fiber, there was not that liability of oozing on to the gum and creating a sloughing, that there was with nerve paste. He used the nerve paste himself, and always cauterized the gum at the septum, with carbolic acid, for fear of sloughing, except when he applied the rubber dam, for such cases, with the view of greater safety.

Dr. Trueman was more disposed to favor the employment of nerve paste, as he then knew just how much he used. He thought nerve fiber was made by steeping cotton floss in "Fowler's solution of arsenic." He had used disks made of asbestos paper charged with arsenic, but had not found them as effective as the paste.

Dr. Cupein could account, in a measure, for the behavior of nerve fiber or asbestos disks not acting as promptly on the pulp, because this substance was not entirely arsenic, but the arsenate of potash. Fowler's solution is not arsenic pure and simple, but really a one per cent. solution of the arsenate of potash; hence, while containing sufficient arsenic to obtund sensitive dentine, as Dr. Register has demonstrated, it may not contain sufficient arsenic to devitalize the pulp. He thought the fiber might be used successfully in conjunction with the paste, instead of employing cotton with this. His mode was to apply the paste and cover this with a disk of lead or paper and on this apply cotton; by using nerve fiber next the paste, and cotton floss over the nerve fiber, he believed good results could be attained. He had been very successful by sealing the nerve paste in with phosphate cement.

Dr. Trueman brought forward the subject of inlays. He had found that it took about seven minutes to fuse the bodies furnished by the Dental Protective Company—in a Downie furnace. He was more disposed to favor high-fusing bodies than low. He had endeavored to make a furnace and use nickel as a muffle. Although nickel is nearly as resistant as platinum, this metal fused as soon as the high-fusing bodies; therefore could not be used as a muffle.

When grinding high and low fusing bodies, the former was cut by the grindstone very much as an artificial tooth, while the low fusing body cut somewhat as a dense sample of oxyphosphate filling material after it had set well. He thought that the low fusing bodies could be used in approximal or labial cavities, but that high fusing bodies were better suited to restore broken corners or on occlusal surfaces.

Dr. Bonsall was able with his Ash's electrical furnace to melt low fusing bodies in three-quarters of a minute.

Several of the members present spoke of the visit of an agent giving instruction for one dollar in the art of soldering aluminum. The solder was composed of six parts of aluminum, two parts of zinc and four parts of phosphor tin; the flux used was stearic acid. The agent demonstrated the soldering of a simple ferrule. The lapped edges were well cleaned and carefully fitted, and the solder made to flow by holding over the flame of an alcohol lamp or the absolutely blue flame of a Bunson burner. The solder is sluggish, but is pushed along the seam by means of a piece of iron wire in the form of a loop.

Dr. Chas. Chupein had made an aluminum crown, but found, when finishing this, that the solder had not flowed at all points as it had appeared to do.

Dr. Theodore Chupein had soldered a loop or attachment to an aluminum plate, such as is used when employing such plates in combination with rubber, but, although the loop held to the plate firmly, the solder had not flowed evenly.

The agent said that an entire set of teeth could be invested and soldered in this way, but, from the behavior and sluggishness of the solder, in the simple cases which he had tried, and the care he had used to have every part clean, he doubted if this could be done.—*Office and Laboratory.*

